# The emotional context of self-management in chronic illness: A qualitative study of the role of health professional support in the self-management of type 2 diabetes

**DOI:** 10.1186/1472-6963-8-214

**Published:** 2008-10-17

**Authors:** John Furler, Christine Walker, Irene Blackberry, Trisha Dunning, Nabil Sulaiman, James Dunbar, James Best, Doris Young

**Affiliations:** 1 Dept of General Practice, University of Melbourne, Carlton, Australia; 2 Chronic Illness Alliance, Melbourne, Australia; 3 School of Nursing, Deakin University, Geelong, Australia; 4 Greater Green Triangle University Department of Rural Health, Flinders University, Adelaide 5001, Australia; 5 Greater Green Triangle University Department of Rural Health, Deakin University, Warrnambool, Victoria, Australia; 6 Faculty of Medicine, University of Melbourne, Carlton, Australia

## Abstract

**Background:**

Support for patient self-management is an accepted role for health professionals. Little evidence exists on the appropriate basis for the role of health professionals in achieving optimum self-management outcomes. This study explores the perceptions of people with type 2 diabetes about their self-management strategies and how relationships with health professionals may support this.

**Methods:**

Four focus groups were conducted with people with type 2 diabetes: two with English-speaking and one each with Turkish and Arabic-speaking. Transcripts from the groups were analysed drawing on grounded hermeneutics and interpretive description.

**Results:**

We describe three conceptually linked categories of text from the focus groups based on emotional context of self management, dominant approaches to self management and support from health professionals for self management. All groups described important emotional contexts to living with and self-managing diabetes and these linked closely with how they approached their diabetes management and what they looked for from health professionals. Culture seemed an important influence in shaping these linkages.

**Conclusion:**

Our findings suggest people construct their own individual self-management and self-care program, springing from an important emotional base. This is shaped in part by culture and in turn determines the aims each person has in pursuing self-management strategies and the role they make available to health professionals to support them. While health professionals' support for self-care strategies will be more congruent with patients' expectations if they explore each person's social, emotional and cultural circumstances, pursuit of improved health outcomes may involve a careful balance between supporting as well as helping shift the emotional constructs surrounding a patient life with diabetes.

## Background

Supporting self-management by patients with chronic conditions is now an accepted and important part of addressing the disease burden and health service use associated with chronic disease in many countries. In this paper we use the term self management to include the full range of activities undertaken by a person with a chronic condition, ranging from preventive activity, undertaken by currently healthy people at home (sometimes known as self-care) through to the day-to-day tasks undertaken by an individual to manage symptoms, treatments, consequences and lifestyle changes associated with a chronic condition [1]. Most people with chronic conditions already see themselves as actively self-managing, drawing on a range of supports in 'doing' the long and often 'hard work' [2] involved in living with a chronic condition. Nevertheless supporting patient self-management is seen as an important task of health services [3].

A wide range of approaches to deliver interventions to enhance and support self-management have been used [1,4,5]. Interventions vary in the use of group or one to one approaches and in the content included in the support intervention. There is a trend towards patient-centred and patient-driven approaches to defining the content of interventions [6]. Nevertheless reviews of self-management support continue to identify a predominant focus on education and advice giving with less attention to the psycho-emotional issues patients face.

The extent of involvement of health professionals is a further key point of difference on which interventions differ. In the past professional-led interventions dominated. In order to give higher priority to patient defined needs, lay or peer-led programs have been developed in the USA [7] and taken up widely in the UK [8] and elsewhere. Yet the benefits of such programs have been questioned [9-11]. The relatively small improvements in health outcomes as well as the small numbers who attend such programs suggest such generic, scripted self-management programs may not be attractive to many people with chronic illnesses, failing to grapple with the complexities and psychosocial and emotional needs involved in making a meaningful life with chronic illness [9,12].

Supporting self management, it has been suggested, is in fact best done through "a trusted [health] professional in the context of routine service delivery rather than through classes" [3]. Integrating self-management support with people's usual source of primary care seems important. Yet this may not address the unmet need for social and emotional support identified in the way self management support has evolved thus far. Health professionals may have concerns about the very notion of self-management and its aim of encouraging patients' autonomy and control over their health and health care at a time when professionals are increasingly accountable for disease outcomes and adherence to evidence based guidelines. Health professionals may not be well trained and prepared for effectively supporting self care. In this context they tend to continue to focus on advice giving and education with less attention to underlying psychological and social issues [13]. Indeed concerns have been expressed that health professionals may even devalue or disrupt important social supports that people draw on to self-manage their condition [3,14-16]. Clearly getting the balance right for the role health professionals play in supporting self-management is complex but important.

Given this uncertainty over the role of health professionals in supporting self-management, researchers in the Patient Engagement and Coaching for Health (PEACH) study (see below) undertook focus groups to explore the perceptions people with type 2 diabetes have of the role health professionals play in supporting them to improve their self-management strategies. The PEACH study involves General Practice based practice nurses "coaching" patients by phone about their diabetes [17]. The intervention is led by practice nurses and specifically encourages and supports the patient to be more active and engaged in managing their diabetes and in their relationship with their family medical practitioner (General Practitioner or GP).

Our aim in this focus group study was to ensure that our coaching intervention would strengthen the positive role and potentially supportive relationship of primary care health professionals within the context of people's lived experience of self-management of diabetes. We specifically explored people's perceptions about the role played by doctors, practice nurses and other health professionals within an individual's overall social support that they draw on in their diabetes management and how relationships with health professionals influence self-management. In this paper we report findings from this exploratory study and suggest important implications for health professionals who support people with type 2 diabetes in their self-care.

The study is located in a socioeconomically and culturally diverse locality of outer urban Melbourne in Australia. The area has high levels of socio-economic disadvantage and a high proportion of the population speak languages other than English at home, mainly Turkish and Arabic [18]. In Australia type 2 diabetes is more common in both socioeconomically disadvantaged groups [19] and the Turkish and Arabic born communities [20]. Socioeconomic disadvantage and cultural difference thus both formed important contexts for our study. Previous work undertaken in this area by members of our team with Turkish and Arabic-speaking communities highlighted the importance of social and cultural context in the perceptions people have of diabetes and their potential to make behaviour changes [21].

## Method

### The focus groups

Our interest was in diabetes as a socially enacted and lived experience. We wanted to understand the way clinical interactions may or may not support self-management enacted as a day-to-day lived experience subject to the "shared frames of meaning" [22] that surround people with diabetes in their communities. Focus groups can help show how social processes, norms and values are at play in the shaping of health behaviours. We wanted to generate data that would help us ensure the content of our telephone coaching intervention most effectively engaged with and accounted for these underlying notions. While not looking to generate "truths" about how people actually self-manage their diabetes (nor to define particular ideal ways of self-managing to achieve well controlled diabetes), focus groups allowed us to understand the ways that people were prepared to understand and discuss their diabetes within a public, social interactional setting, which is important for shaping our intervention.

### Recruitment of participants

Four focus groups were conducted over the first half of 2006. Participants were people with Type 2 diabetes living in the study target area. Consistent with our research design and interest, we did not set out to recruit people with either well or poorly controlled disease. Recruitment took place through a community health service and ethnic support groups. We used a database of people who had at least one previous contact with a diabetes educator based at the community health service to identify potential participants who were then approached by letter or through personal contact. Fifty two people in total (24 women, 26 men) were involved in four separate focus groups, most aged between 50 and 80. Most were either unemployed or retired. Duration of diabetes ranged from 1 – 16 years. We held two groups in English, for patients from a range of backgrounds (including Anglo Celtic but also other ethnic backgrounds) and one group each in Turkish and Arabic, facilitated in English with an interpreter assisting, to ensure we included patients from these two largest non-English speaking communities in the study area. Each group met once and the session lasted approximately 1.5 hours. Ethics approval for the study was obtained through the University of Melbourne Human Research Ethics committee.

### Data collection and analysis

Discussion in the groups explored how participants currently care for themselves, barriers and enablers to self-management they experience, as well as their relationship with health professionals and how that influences the self-management strategies they adopt. Central concepts explored were the role of self-efficacy and personal control and how relationships and interactions with health professionals influenced these (see Additional file 1). The sessions were led by one of the investigators (CW) who is experienced in the use of focus group in social health research. The Arabic speaking dietician known to the local community who had assisted with recruitment sat in on the groups. Interpreters in the non-English speaking groups were qualified interpreters with experience in health settings and familiar to the local community. We made efforts to include all participants in the group discussion in all of the groups. Each session was audiotaped and then transcribed verbatim. In all groups, transcriptions were made in English (using the interpreter's real-time translation in the Turkish and Arabic speaking groups). While Knodel [23] notes ideally that data should be transcribed in both the language of the participants and a common language, due to cost and time constraints it was not possible to use this technique. Other limitations related to the methods used in the study are discussed below.

Our approach to analysis is broadly interpretive. We bring our existing interests and a range of clinical and research knowledge of the field. This was primarily focused on practitioner and health system perspectives of improving quality of care in diabetes, and a particular interest in structural barriers to self-management. One of our research team is an experienced consumer researcher across the field of chronic illness. We nevertheless tried to retain openness to points of departure from our existing interest. We wanted to produce an interpretation that would resonate with and offer opportunities for application in the real world of diabetes self-management and clinical practice. In this we draw on emerging traditions of analysis within applied social health research including grounded hermeneutics [24] and interpretive description [25]. Analysis of the focus group transcripts was undertaken separately by two of the researchers (CW and JF) to enhance validity of our identified themes. They compared and discussed theme lists to look for important similarities and resolve differences. The results are reported here are focused on three linked categories that provide an important insight into the phenomenon of diabetes self management and the role that health professionals can play in supporting that.

## Results

### The emotional context of living with diabetes

Emotional responses were prominent in talk about living with and self-managing diabetes across all groups. Throughout, the group discussions were peppered with expressions of "shock" at the diagnosis, of fear of the future, or of the important need to deal with emotional highs and lows as a part of self-managing diabetes. In the English-speaking groups this was underpinned by participants' acknowledgement of the ongoing challenge involved in sustaining strategies of self-care, because of the very nature of diabetes:

"It's for a lifetime so we have to care for ourselves and we can't slack off anywhere because we are going to pay for it; you can't start eating chocolate"

This recognition that neglect would result in deteriorating health was accompanied by a sense of resentment that many enjoyable aspects of life had been removed, and that they had been unjustly singled out by this disease. This is seen in this sequence of comments from the group;

"I did get a shock [...]

I wondered where I got it from [...]

I was looking for an answer too [...]

So I think why did I get this [...] Yes you do the right thing all your life, don't drink or smoke [...] It's like you miss out on all the good things."

The sense of injustice was accompanied by anxiety and uncertainty about their future life with diabetes and a distrust of their bodies as a result of the diagnosis, refected in comments such as:

"it made me worry about the future and how to control it"

and

"I do worry about long term effects because I know you don't feel things until they are permanent"

Emotional responses were also prominent in the Turkish and Arabic-speaking groups. Rather than resentment and anxiety, here participants spoke predominantly of the need to manage their daily lives to avoid or minimise stress. Being subjected to stress and becoming upset, it was asserted, would override all health interventions and cause a person to become ill independently of other factors such as diet. Serious stress could cause diabetes, and in particular some participants in the groups linked diabetes to critical distressing life events:

'After my husband passed away, about a year after I got diabetes. I was stressed.'

and

"I believe that getting upset brings diabetes, because one week before I heard about my mum (becoming unwell) I didn't have diabetes"

Becoming upset and stressed could also worsen ongoing control of diabetes, in a way that seemed at least as important as behaviours such as diet and exercise:

'If I'm normal my sugar levels in my blood are good, but when I get very upset and angry it just jumps higher.'

and

When you stress yourself and your morale's (sic) are down, I'm quite sure it'll come out. When my morale's are really good and up my sugar levels are really good, not too bad

### Approaches to self-management

We focus on lifestyle behaviours here. Talk about the approach people took to managing their condition was closely linked to this emotional context of living with diabetes. For example, consistent with the resentment seen in the English-speaking groups, participants saw managing their diabetes as a process of coping with loss. People spoke of what they went without rather than what they currently enjoyed eating, of loss rather than of adopting healthy eating habits. One participant had to "modify the way we eat and just get on with it", another decried that "it stopped me eating chocolate", while another noted that he and his "shouldn't be eating that – we have cut out butter" Linked to this 'deficit' approach to modifying diet was the discipline that these participants spoke of bringing to their diabetes. Lifestyle behaviors became a focus and a tool for "staying on the straight and narrow". While living with diabetes gave you a "reality check" or "sort of reins you back to reality", nevertheless

"*you can readjust and try and do what's the normal thing to care for your body"*

So life became a matter of "staying on top of it (the diabetes)" and each individual was responsible for themselves, as seen in this sequence of comments:

"You have to be accountable for your own actions

[...]

You have to look after yourself or your life won't be long

You don't rely on someone else to do it for you [...]

If you don't look after yourself, no-one else will do it for you"

Adjusting through individual will and discipline was a way of controlling the disease and thus reducing anxiety and uncertainty:

"when S said 'what about the future?' I realised I was worried about the future and I need to stay away from lollies my wife buys and get out and exercise more and stay on a diet."

In common with findings elsewhere [26], "strategic non-compliance" or occasional dietary indiscretions as the other side of ongoing self-discipline was commonly reported across the English speaking groups.

Where stress was the dominant emotional response, as seen in both Turkish and Arabic speaking groups, self-managing diabetes was described more as a process of staying calm. Lifestyle behaviours were present but not a sole focus, sitting alongside and closely entwined with, attending to ones emotional balance, seen in this sequence of comments from the Turkish group:

"I need to look after my diet, yes, and also keep myself happy, my morals up and keep the stress away [...]

... For me, a lot of walking, watch what I eat, and not to get myself upset [...]

... Not to get stressed [...] I drink a lot of black tea [...] if I don't get hooked on worries and things I'm fine"

It seemed hard to separate information, practical help and emotional support in this way. For one woman:

"My husband looks after me and he tells me what's right to do for diabetes [...] and because getting upset is not good for diabetes, so he helps me for entertainment and amusement and stuff that helps me"

In this context talk about diet was constructed in a more positive way. For example substituting grilled and fresh foods for fried foods, using 'good olive oil' instead of poor quality oils were all examples of positive dietary changes which the groups enjoyed pursuing.

Physical activity was less a focus of talk in the groups than dietary changes. All the groups reported walking for exercise, both with others and alone, often because of poor access to other forms of physical activity, such as not being able to afford gymnasiums, or access swimming pools.

### Health professional support for self-management

All focus groups acknowledged that a relationship with a health professional was important and helped them to self-manage their condition. The way groups discussed being supported (or not) through relationships with health professionals was again linked to the emotional states accompanying life with diabetes and infusing the approaches people took to self management. Where self discipline and disease control were the focus of self management, as in the English speaking groups, information was paramount. Here it was important to "shop around and get the right one (GP) to get the right answers". People were prepared to change doctors or use a range of other health services to get the information they wanted to enable them to exert control over their condition. For one participant the "GP gave me some very basic advice but the best thing he did for me was to refer me here to the dietician". Where control was the desired outcome, the doctor became an active player in generating the emotional context that infused that. In this sequence of comments, we can see that even if diabetes control seems adequate, in the pursuit of ever tighter control the relationship with the doctor can create a powerful accompanying emotional tone:

"My doctor upset me because my morning readings are always high [...] when I say high I mean in the 7's and I was told that if it was under 8 it was OK [...]

*My doctor upset me too, he wanted it down below 6 [...]*.

...I'm a vegetarian and I thought what else can I cut out? [...] I don't think he should have frightened me..."

In this context, for the English-speaking groups the central relationship was not necessarily with their GP. They described a choice of consulting with dieticians, diabetes nurse educators, podiatrists or nurses. They spoke warmly of the services they received from the health professionals at the local Community Health Service, most particularly education sessions where they learnt to read food labels.

"It was a real support,...you learnt things you couldn't get elsewhere"

They often chose one person from the range of health professionals, with whom they formed a personal bond, seeking advice from that person or contacting them if they were anxious, while still keeping appointments with the other health professionals involved in their care. For the English-speaking group, referral to the Diabetes Nurse Educator or dietician by their GP gave them access to information and an opportunity to regain some control:

*"I found that I came here straight away and got under the DNE's [diabetes nurse educator] umbrella like and got education about how to read labels and that was more beneficial than any doctor at the time"*.

Where the desired outcome seemed to be to be primarily focused on maintaining a sense of calm and avoiding stress, as seen in the Turkish and Arabic-speaking groups, there was a tendency to look for reassurance in the relationship with the doctor and focus particularly on that relationship. Information was important but did not seem to be constructed as a tool for discipline and subsequent success or failure:

"The doctor usually tells you everything you need to know"

These participants were still engaged with other health professionals, but often at the behest of their GP and this seemed to create a sense of containment. When asked who the best person to help with diabetes was, the doctor is primary and other professionals and resources flowed from that, seen in this sequence of comments:

*"The doctor*.

The doctor. And we got information from the computer [...] books

*The doctor sent us here (the community health centre) to get information*.

My doctor..."

This reliance on the GP was not always because their GP spoke their language as many saw English-speaking GPs using relatives or interpreters to assist during consultations. Across the Turkish and Arabic-speaking groups, discussion reflected a sense of contentment with these arrangements:

*"Basically we go to the doctor and the hospital"*.

There was a sense in the Turkish and Arabic-speaking groups that people would not have attended any education sessions if the GP had not strongly recommended they do so. In this context, the GP offered more than technical support and information. They valued the sense of caring they received from the GP which was critical to engendering a sense of emotional calm around living with diabetes:

"As long as there is treatment and people take care of you, I should get used to what I have"

and

"Its not at fault to say I am sick, what's at fault is not to look after the sick person"

It is worth noting that, although cultural difference is central to the range of responses we have reported here, family supports networks, including children and grandchildren were also important across all groups, and in particular such family supports were clearly shaped by gender, as noted in other studies [27,28]. Men across all groups acknowledged their wives had usually adopted the supporting role, learning about food product labels, shopping for the right products and cooking appropriate meals. Women did not tend to receive this level of support. Women already caring for the dietary needs of husbands with diabetes or heart conditions simply adopted the diet and exercise regime themselves when they were diagnosed with diabetes. Interestingly, in the English speaking groups the role of partner support was enlisted in the quest for disease control through discipline:

"...I think it would be terrific if there were courses set up especially for partners [...] my wife is not unsupportive [...] but I don't think they understand the temptation they put in front of you..."

## Discussion

Our results suggest that living with and self-managing diabetes is as much a social and emotional task as a technical task. Social circumstances and emotional responses infuse the perceptions people have of their condition and this is linked to the way they care for and manage it, particularly in relation to diet and physical activity. This in turn has implications for the role health professionals can play in supporting patients' self-care.

Emotional states and their link to health are an important focus of research into health care from a sociological perspective [29] as well as increasingly seen in clinical research [6,30]. Thorne and Patterson [30] explored how understanding the emotional needs of a patient can be an important element of ensuring that health professionals provide appropriate support to patients over the evolving trajectory of living with an illness such as diabetes. What our study offers is reinforcement that emotional states potentially form a critical context and element of any patient's approach to managing their diabetes as well as a tentative interpretation [25] showing how cultural context may be important within that.

Recently much has been made of how the context of increasing bureaucratisation of health care work with layers of accountability to funding and professional bodies has focused attention on surveillance and monitoring of patients and their disease and how this plays a role in shaping notions of 'patienthood' [31,32]. Macdonald et al [33] note how practice nurses in primary care in adapting to this changing work context develop heuristic notions of patients as "good self managers and bad self managers", allowing this to dictate and anticipate illness trajectories. While our findings do not link particular responses to disease outcomes or diabetes control, nevertheless they do provide insight into the way patients interact with these concepts identified elsewhere.

One response we identified, seen mainly in the English-speaking groups, saw diabetes as an unjust and unwarranted imposition on their lives. They looked to health professionals and self-care strategies to help wrest back control. They chose from a 'smorgasbord' of available services, using health professionals to achieve this. Both family members and health professionals played a role in assisting them gain confidence and a sense of personal control over their diabetes, particularly where healthy eating was concerned. Another response, seen mainly in the Turkish and Arabic-speaking groups, saw diabetes as yet another thread in the fabric of life, where health was a product of the conditions of existence, in constant play with internalised levels of stress. Doctors, in taking some responsibility for the patient's diabetes and in providing a trusting, reassuring presence, played an important role in reducing stress. Diabetes control seemed a secondary consequence. What is important is to see how the former scenario more naturally feeds into the idealised form of 'patienthood' currently evolving through the professional context of care. What is also interesting to note is that the patients participating in this study seemed to indicate that their relationship with their doctor in particular had adapted to their own emotional illness context.

Our study simply highlights these emotional domains as important areas of engagement between GP and patient in the context of a long illness such as diabetes. Whether health professionals must acquiesce with or subtly challenge or even disrupt a patient's emotional context to their illness is not clear. While our study resonates with notions of patient centeredness and autonomy, now thought to be central to productive and effective patient-professional relationships and improved health outcomes, it does highlight the potential harm that may flow from an uncritical focus on the primacy of the doctor-patient relationship [34] and its potential to foster particular cultural stereotypes that may even shut down particular illness trajectories and improved health outcomes.

### Limitations and strengths of the study

One strength of this study has been the way the focus group interactions can be seen at work through the sequences of text reported here, showing the way social processes directly shape group based understandings of illness. Nevertheless there are a number of important limitations to this study. A number of power differentials may have been operational in a way that put limits on the group discussion. The groups were conducted with some members of the research team present and with the dietician (with whom members of the groups had previously had contact and through whom recruitment had been initiated) present in the group. This could have significantly influenced the comfort group members felt to contribute openly to the group discussion, as well as shaping the sorts of responses they felt able and willing to offer. We attempted to deal with this by creating an ambience within the group that was as warm and open as possible. Food and beverages were served, and an informal atmosphere was encouraged, research team members took positions to the back of the room, organised chairs, refreshments etc in an attempt to have them seen as supportive facilitators of the group rather than external judges of the group discussion. The dietician openly acknowledged that she was interested in all the views of the participants, including any that may be critical of support services available. Cultural norms may themselves have set limits on the group discussions. In some cultures elder or more senior or dominant persons from within a local community may have precedence, and members in a less powerful position may not feel able to make contradictory comments. Responses might also have been different if the groups had been conducted entirely in the participants own language [35]. We attempted to deal with these influences by actively trying to engage all group members in the discussion. Nevertheless such limits may well have been at play. However we felt that this would not necessarily lessen the relevance of the data as such family and culturally based limits will form an important part of the context any person with diabetes must work within as they self-manage their condition away from immediate health services and it was this domain of lived diabetes experience that we were particularly focused on. Finally, stemming from these limitations, the findings of our study cannot be generalised beyond the communities of our study area. Exploratory work of this nature aims rather to show phenomena seen in real world practice and offer only tentative implications for the role played by health professionals in supporting patients in self-management and self-care of diabetes, as well as for the design and implementation of diabetes self-management programs. Our focus is primarily on cultural difference and we have not explored in detail other factors such as gender and SES, although gender has been found in the past to shape patients perceptions of their diabetes [27,28].

## Conclusion

Self-management support does need to be embedded within an ongoing relationship with a trusted health professional but a one size fits all approach will not do. Our findings suggest that, for health professionals, an important way of supporting patients' efforts at self-managing diabetes is through engagement with the emotional context within which patients understand and live with their condition. Health professionals need to work with patients in a way that is consistent with how they integrate self-care into their everyday lives, constructing and co-ordinating their own "self-management program". Nevertheless looking for ways to restructure or reinterpret this emotional context may be an important avenue for patient and health professional to move forward. Critical self-reflection by health professionals will be important here to avoid undermining or devaluing the complex web of supports they draw on living with diabetes. Future research could well focus on the qualities of patient-professional relationships that allow flexibility, questioning and change over time and how this relates to longer term health outcomes.

## Competing interests

The authors declare that they have no competing interests.

## Authors' contributions

All the authors contributed to conceptualising the study. CW and JF finalised the study design. CW, IB and JF conducted the focus groups. CW and JF analysed the data. CW drafted the first version of the paper. JF led revisions of the paper. All authors contributed to writing the paper and read and approved the final manuscript.

## Pre-publication history

The pre-publication history for this paper can be accessed here:



## Supplementary Material

Additional file 1 **Focus group questions.** Click here for file
