# Supplementary material for: The emotional context of self-management in chronic illness: A qualitative study of the role of health professional support in the self-management of type 2 diabetes
Source: BMC Health Serv Res. 2008 Oct 17;8:214. doi: 10.1186/1472-6963-8-214 (PMC2596123; doi:10.1186/1472-6963-8-214)
Supplement: Additional file 1 — Focus group questions. [file 1472-6963-8-214-S1.doc]

**Focus Group Questions**

Tell us about the main way diabetes has affected your life?

What are your main sources of help for your diabetes?

- What makes it easy for you to use them?
- What makes it more difficult for you to use them?

What other possible sources of help for diabetes are you aware of that you don’t currently use?

- Why don’t you use them?
- How could they be changed to make them more useful for you?

What have been the best options for help for you for your diabetes in the long term?

- What has helped you stay involved with that person/service/source of help?

What is it like going to your GP to have your diabetes looked after?

- Have you ever felt you would like to have more say in your diabetes care when talking with your GP?
- How do you think you or other people with diabetes in general could have a greater say in their diabetes care?
- How could they work with their GP to have more say in their care?
